# Therapeutic Benefit of Top-Selling Oncology Drugs in Medicare

**DOI:** 10.1001/jamanetworkopen.2025.3323

**Published:** 2025-04-04

**Authors:** Stephanie Wang, Alexander C. Egilman, Georg Hahn, Aaron S. Kesselheim

**Affiliations:** 1 Harvard College, Cambridge, Massachusetts; 2 Program on Regulation, Therapeutics, and Law, Division of Pharmacoepidemiology and Pharmacoeconomics, Department of Medicine, Brigham and Women’s Hospital and Harvard Medical School, Boston, Massachusetts

## Abstract

This cohort study examines whether international health technology assessment reviews may identify oncology drugs that are suitable for aggressive price negotiation.

## Introduction

Cancer drugs cost $74 000 more, on average, than noncancer drugs,^1^ contributing to patient financial toxicity. In the US, drug manufacturers can set and increase prices during periods of market exclusivity lasting approximately 13 to 17 years.^2^ The Inflation Reduction Act of 2022 authorized Medicare to negotiate prices for top-selling brand-name drugs after 9 years (13 for biologics) based partly on their therapeutic benefits compared with alternatives. International health technology assessment (HTA) reviews may help identify drugs that do not offer clinical benefits over competitors and might be suitable for aggressive price negotiation.

## Methods

In this cohort study, we analyzed the 50 top-selling brand-name oncology drugs in Medicare in 2022 according to combined prerebate spending in Medicare Part B and Part D using publicly available data. Postrebate spending for Medicare Part D drugs was estimated using sales-weighted, non-Medicaid, 4-quarter rolling average rebates from SSR Health; we assumed the class average rebate for oncology drugs of 9.5% when estimates were not available.^3,4^ We used Eversana NAVLIN’s database to determine ratings of added therapeutic benefit through 2022 from Germany’s Federal Joint Committee and France’s Transparency Committee; these ratings are independent of price.^5^ Drugs receiving ratings of considerable, major, important, or moderate were categorized as high added benefit; other ratings were considered either low or no added benefit.^6^ Drugs were categorized according to their most favorable rating across countries, dosages, subpopulations, and indications. Data analysis was performed using Excel software version 16.84 (Microsoft) and R statistical software version 4.3.1 (R Project for Statistical Computing). This study was conducted in accordance with the STROBE reporting guidelines for cohort studies. Informed consent and institutional review board approval were not needed because no patient data were used, in accordance with 45 CFR §46.

## Results

Thirty-one drugs (62%) were small molecules, 27 (54%) were primarily reimbursed by Medicare Part D, and 44 (88%) were approved for at least 1 rare cancer. Forty-eight drugs (96%) received at least 1 rating from Germany or France, with 39 (81%) classified as high added benefit, 8 (17%) as low added benefit, and 1 (2%) as no added benefit (Table 1).

**Table 1.** Top 50 Selling Oncology Drugs in Medicare in 2022 by Added Therapeutic Benefit Ratings

Brand name^a^	Generic name	Primary Medicare program	Single source drug	Total Medicare spending, $ (billions)		Rank^b^	Total Medicare beneficiaries	Estimated net annual spending/beneficiary, $	Added therapeutic benefit rating		
				Prerebate	Net				Overall	Germany	France
Keytruda	Pembrolizumab	B	Yes	4.94	4.94	6	67 022	73 647	High	High	High
Imbruvica	Ibrutinib	D	Yes	2.85	2.53	13	22 202	114 018	High	High	High
Ibrance	Palbociclib	D	Yes	1.95	1.75	22	17 497	100 217	Low	None	Low
Opdivo	Nivolumab	B	Yes	1.85	1.85	23	26 957	68 626	High	High	High
Ofev	Nintedanib	D	Yes	1.76	1.6	26	20 686	77 129	High	High	Low
Jakafi	Ruxolitinib	D	Yes	1.76	1.49	27	13 486	110 703	High	High	High
Darzalex Faspro	Daratumumab hyaluronidase-fihj	B	Yes	1.58	1.58	32	17 976	87 693	High	High	High
Tagrisso	Osimertinib	D	Yes	1.08	0.9	41	8629	104 381	High	High	High
Calquence	Acalabrutinib	D	Yes	1.03	0.95	42	11 181	84 815	High	High	NA
Cabometyx	Cabozantinib	D	Yes	0.92	0.67	48	7315	91 738	High	NA	High
Tecentriq	Atezolizumab	B	Yes	0.78	0.78	63	12 812	60 705	High	High	High
Venclyxto	Venetoclax	D	Yes	0.76	0.69	65	17 540	39 088	High	High	High
Lenvima	Lenvatinib	D	Yes	0.63	0.62	86	7066	87 084	High	High	High
Rituximab	Rituximab	B	No	0.58	0.58	94	27 486	21 071	High	High	High
Imfinzi	Durvalumab	B	Yes	0.56	0.56	98	10 517	53 508	High	High	High
Sprycel	Dasatinib	D	No	0.5	0.42	109	5182	81 128	High	NA	High
Lynparza	Olaparib	D	Yes	0.46	0.43	116	6026	71 027	High	High	Low
Alimta	Pemetrexed	B	No	0.45	0.45	125	16 239	27 465	High	NA	High
Yervoy	Ipilimumab	B	Yes	0.44	0.44	127	7733	57 372	High	High	Low
Inlyta	Axitinib	D	Yes	0.42	0.33	138	4737	69 729	High	High	Low
Tasigna	Nilotinib	D	Yes	0.38	0.35	152	3024	116 006	High	NA	High
Kyprolis	Carfilzomib	B	Yes	0.37	0.37	159	5990	61 227	High	High	High
Ninlaro	Ixazomib	D	Yes	0.34	0.28	176	3985	70 524	Low	Low	None
Taxol	Paclitaxel	B	No	0.32	0.32	183	15 213	21 308	High	NA	High
Darzalex	Daratumumab	B	Yes	0.32	0.32	188	4961	64 164	High	High	High
Velcade	Bortezomib	B	No	0.31	0.31	192	15 807	19 388	High	NA	High
Brukinsa	Zanubrutinib	D	Yes	0.3	0.27	195	3891	70 549	None	None	NA
Gleevec	Imatinib	D	No	0.26	0.2	213	14 653	13 793	High	NA	High
Enhertu	Trastuzumab deruxtecan	B	Yes	0.26	0.26	217	4841	53 269	NA	NA	NA
Avastin	Bevacizumab	B	No	0.25	0.25	222	140 895	1792	High	NA	High
Kesimpta	Ofatumumab	D	Yes	0.23	0.2	236	3418	57 407	High	NA	High
Votubia	Everolimus	D	No	0.22	0.2	244	5714	35 577	High	NA	High
Libtayo	Cemiplimab	B	Yes	0.2	0.2	258	3187	63 957	Low	Low	NA
Padcev	Enfortumab	B	Yes	0.19	0.19	274	1910	99 122	High	High	NA
Adcetris	Brentuximab	B	Yes	0.19	0.19	277	1569	118 681	High	Low	High
Kadcyla	Trastuzumab emtansine	B	Yes	0.17	0.17	292	2822	60 438	High	Low	High
Bosulif	Bosutinib	D	Yes	0.17	0.16	294	1628	97 543	Low	Low	None
Kisqali	Ribociclib	D	Yes	0.17	0.15	304	2108	71 335	Low	Low	None
Calquence	Acalabrutinib maleate	D	Yes	0.16	0.15	309	5519	27 461	High	High	NA
Mekinist	Trametinib	D	Yes	0.16	0.14	319	2281	63 111	High	High	High
Trodelvy	Sacituzumab	B	Yes	0.16	0.16	325	2120	73 719	High	High	High
Herceptin	Trastuzumab	B	No	0.16	0.16	327	4520	34 367	High	NA	High
Zejula	Niraparib	D	Yes	0.15	0.13	329	2211	57 385	Low	Low	Low
Erbitux	Cetuximab	B	Yes	0.15	0.15	331	3695	41 665	High	NA	High
Alecensa	Alectinib	D	Yes	0.15	0.1	332	1284	78 285	Low	Low	Low
Jevtana	Cabazitaxel	B	Yes	0.14	0.14	342	2919	49 654	High	NA	High
Lonsurf	Trifluridine and tipiracil	D	Yes	0.14	0.1	345	2710	35 841	Low	Low	None
Gazyva	Obinutuzumab	B	Yes	0.14	0.14	350	4364	31 787	High	Low	High
Tafinlar	Dabrafenib	D	Yes	0.14	0.09	353	2171	39 503	High	High	NA
Tibsovo	Ivosidenib	D	Yes	0.13	0.12	359	971	125 622	NA	NA	NA

Abbreviation: NA, not available.

^a^ Drugs were classified as cancer treatments based on the World Health Organization Anatomical Therapeutic Classification system, specifically those under category L01 (antineoplastic agents), and had at least 1 approved cancer indication.

^b^ Refers to rank among overall top-selling Medicare drugs.

In 2022, Medicare prerebate spending for the 48 drugs totaled $31.3 billion, or $29.1 billion after subtracting estimated rebates (Table 2). Estimated postrebate Medicare spending totaled $26.0 billion for the 39 high-added-benefit drugs, $2.9 billion for the 8 low-added-benefit drugs, and $275 million on the 1 no-added-benefit drug. Median (IQR) postrebate spending per beneficiary was $61 227 ($37 332-$82 971) for high-added-benefit drugs, $70 929 ($62 314-$83 099) for low-added-benefit drugs, and $70 549 ($70 549-$70 549) for the no-added-benefit drug.

**Table 2.** Medicare Use and Spending on Top-Selling Oncology Drugs in 2022

Variable	Received health technology assessment rating (N = 48)	High added benefit (n = 39)	Low added benefit (n = 8)	No added benefit (n = 1)
Total annual prerebate Medicare spending, $ (billions)	31.3	27.8	3.3	0.3
Total annual prerebate Medicare spending on 50 top-selling oncology drugs in 2022, %	98.8	87.5	10.3	0.96
Total net (postrebate) Medicare spending, $ (billions)	29.1	26.0	2.9	0.3
Total annual net (postrebate) Medicare spending on 50 top-selling oncology drugs in 2022, %	98.7	88.1	9.7	0.93
Prerebate Medicare spending per drug, median (IQR), $ (millions)	321.2 (169.1-658.9)	376.0 (187.8-767.7)	168.2 (154.5-238.0)	303.3 (303.3-303.3)
Estimated net (postrebate) Medicare spending per drug, median (IQR), $ (millions)	312.4 (158.2-629.3)	350.8 (187.8-678.3)	154.6 (120.3-223.1)	274.5 (274.5-274.5)
Total No. of beneficiaries	592 858	554 357	34 610	3891
No. of beneficiaries per drug, median (IQR)	5616 (2998-14793)	7066 (4030-15 510)	2460 (1988-3386)	3891 (3891-3891)
Prerebate Medicare spending per beneficiary, median (IQR), $	69 181 (48 038-89 440)	64 164 (40 488-88 371)	82 197 (68 475-106 165)	77 955 (77 955-77 955)
Estimated net Medicare spending per beneficiary, median (IQR), $	64 061 (39 399-82 050)	61 227 (37 332-82 971)	70 929 (62 314-83 099)	70 549 (70 549-70 549)

## Discussion

This cohort study found that four-fifths of top-selling US cancer drugs provide high added therapeutic benefits according to HTA agencies in France and Germany, and the most effective cancer treatments also earned a majority of revenues. However, cancer drugs offering low or no added benefits accounted for $3.1 billion in postrebate Medicare spending in 2022 and cost more per beneficiary than high added benefit drugs, suggesting opportunities for better aligning clinical benefits and prices of several top-selling cancer drugs. Factors contributing to the widespread use of low-value medications include incentives for pharmacy benefit managers to steer patients to higher-cost drugs, prescribers’ lack of awareness of drug costs, and direct-to-consumer advertising.

Limitations include classifying drugs according to their most favorable rating, thereby potentially misclassifying undeserving drugs as high added benefit. The analysis was restricted to ratings published through 2022; new evidence may lead to different future ratings. Some drugs in our cohort were not eligible for price negotiation. The US should establish a national HTA agency to assess drug benefits and promote value-based pricing. Until then, ratings from experienced HTAs (eg, France and Germany) can help inform Medicare price negotiations, including the identification of low-added-benefit drugs that could be candidates for price reductions owing to the availability of suitable alternatives.

## Notes

Supplement.
Data Sharing Statement
