# Errors in Results and Table 1

**DOI:** 10.1001/jamanetworkopen.2025.39809

**Published:** 2025-10-01

In the Research Letter titled “Therapeutic Benefit of Top-Selling Oncology Drugs in Medicare,”^1^ published April 4, 2025, a coding error related to aflibercept (Zaltrap) resulted in the misattribution of Medicare spending data labeled as *aflibercept* but corresponding to Eylea, an ophthalmologic drug, as being related to Zaltrap, a cancer drug that shares the same active ingredient. As a result, the row for Zaltrap in the published version of Table 1 reflected Medicare ophthalmology use and spending. After the coding error was corrected, Zaltrap no longer qualified for the cohort as 1 of the top 50 cancer drugs by prerebate Medicare spending and was replaced by ivosidenib (Tibsovo). The authors have explained these errors in a Comment,^2^ and this article has been corrected.^1^
