# Supplementary material for: Therapeutic Benefit of Top-Selling Oncology Drugs in Medicare
Source: JAMA Netw Open. 2025 Apr 4;8(4):e253323. doi: 10.1001/jamanetworkopen.2025.3323 (PMC11971663; doi:10.1001/jamanetworkopen.2025.3323)
Supplement: Supplement. — Data Sharing Statement [file jamanetwopen-e253323-s001.pdf]

Data Sharing Statement

Wang. Therapeutic Benefit of Top-Selling Oncology Drugs in Medicare. *JAMA Netw Open*. Published April 04, 2025. doi:10.1001/jamanetworkopen.2025.3323

Data
Data available: No

Additional Information
Explanation for why data not available: Study uses publicly available data.
